# Impact of New Diffusion-Weighted MRI Lesions Following Carotid Artery Stenting on Long-Term Stroke Recurrence

**DOI:** 10.7759/cureus.92031

**Published:** 2025-09-11

**Authors:** Elifnur Kivrak, Ozgur Genc, Zeynep Tanriverdi

**Affiliations:** 1 Department of Neurology, Ortadoğu Private Hospital, Adana, TUR; 2 Department of Radiology, Istanbul Aydın University (İAÜ) VM Medical Park Florya Hospital, Istanbul, TUR; 3 Department of Neurology, İzmir Katip Çelebi University, Atatürk Training and Research Hospital, Izmir, TUR

**Keywords:** carotid artery stenting, diffusion-weighted imaging, ischemic cerebrovascular disease, recurrence, risk factors, stroke

## Abstract

Objective

Carotid artery stenting (CAS) has become an established alternative to carotid endarterectomy (CEA). However, new ischemic lesions, detected by diffusion-weighted imaging (DWI), are frequently observed following the procedure, and their prognostic significance remains controversial. This study aimed to evaluate the impact of new DWI lesions following CAS on long-term stroke recurrence in patients who underwent CAS for carotid artery stenosis.

Methods

This retrospective cohort study analyzed 46 consecutive patients who underwent CAS for symptomatic carotid stenosis between January 2008 and December 2015. All participants had a DWI performed before and within 24 hours after the procedure. Patients were stratified into DWI-positive (DWI+) and DWI-negative (DWI-) groups based on the presence of new ischemic lesions. The primary endpoint was stroke or transient ischemic attack (TIA) recurrence during long-term follow-up. Statistical analysis included the Mann-Whitney U test for continuous variables, the Chi-square test for categorical variables, and Kaplan-Meier survival analysis for time-to-event outcomes.

Results

New DWI lesions were identified in 20 of 46 patients (43.5%) following CAS. Of the 46 patients, eight (17.4%) were lost to follow-up; thus, 38 were analyzed (16 DWI+ and 22 DWI-). During a mean follow-up period of 36.5 months (range, 12-60 months), stroke recurrence occurred in 2 of 16 patients (12.5%) in the DWI+ group, while no stroke recurrence was observed in the 22 patients of the DWI- group (p = 0.170). No significant difference was found between groups regarding myocardial infarction development (DWI+: 2/16 (12.5%) vs. DWI-: 3/22 (13.6%), p = 1.000).

Conclusion

New DWI lesions following CAS may serve as a potential risk marker for long-term stroke recurrence, although statistical significance was not achieved in this cohort. These findings suggest that patients with new post-procedural DWI lesions may represent a higher-risk subgroup; however, this hypothesis requires validation in larger, adequately powered cohorts before guiding clinical practice. Validation studies in larger patient cohorts are needed to confirm these preliminary findings.

## Introduction

Stroke represents the third leading cause of mortality worldwide and constitutes the primary cause of permanent disability [1]. Cerebrovascular diseases account for approximately 15% of all deaths in Türkiye, representing a significant public health burden [2]. Extracranial carotid artery disease contributes to approximately 20%-25% of all ischemic strokes, making it a critical target for prevention strategies [3,4].

Carotid endarterectomy (CEA), long considered the gold standard for carotid stenosis treatment, has demonstrated efficacy in stroke prevention through randomized controlled trials [5,6]. However, carotid artery stenting (CAS) has emerged as an alternative therapeutic option, particularly for patients at high surgical risk [7,8]. The Carotid Revascularization Endarterectomy versus Stenting Trial (CREST) demonstrated equivalent long-term efficacy between CAS and CEA in symptomatic patients [9].

Despite offering a minimally invasive approach, CAS carries procedural complication risks, including periprocedural stroke, myocardial infarction, hemodynamic instability, and distal embolization [10,11]. Distal embolization of atherosclerotic plaque particles during the CAS procedure remains inevitable, despite routine utilization of embolic protection devices [12].

Diffusion-weighted magnetic resonance imaging (DWI) represents an extremely sensitive technique for detecting acute ischemic brain lesions [13]. Studies examining DWI following CAS demonstrate new ischemic lesions in 50%-70% of patients [14,15]. Although most lesions remain asymptomatic, their clinical significance and prognostic value remain subjects of debate.

Evidence on the long-term prognostic value of post-CAS DWI lesions is mixed, ranging from increased risk in the International Carotid Stenting Study (ICSS)-MRI substudy [16] to no association at 12.5-year follow-up [17].

Limited data exist regarding the prognostic value of DWI lesions following carotid revascularization in the Turkish population. Existing literature predominantly contains data from European and North American populations. Due to genetic, environmental, and lifestyle differences, the direct applicability of these findings to the Turkish population remains uncertain [18,19].

The primary hypothesis of this study is that new DWI lesions developing after CAS serve as risk markers for long-term stroke recurrence in the Turkish population. Our primary objective was to investigate the effect of new DWI lesions developing after the procedure on long-term stroke/transient ischemic attack (TIA) recurrence in patients who underwent CAS for symptomatic carotid artery stenosis.

## Materials and methods

Study design and ethical considerations

This retrospective cohort study was conducted as a single-center investigation in the Department of Neurology at a tertiary university hospital. The study was designed and conducted in accordance with the principles outlined in the Declaration of Helsinki and followed the Strengthening the Reporting of Observational Studies in Epidemiology (STROBE) guidelines for cohort studies. The local ethics committee reviewed the study protocol and determined that formal institutional review board approval was not required for this retrospective analysis of routinely collected clinical data, in accordance with institutional policy for retrospective studies. All patient data were anonymized and handled in compliance with patient confidentiality requirements.

Patient selection

A total of 46 consecutive eligible patients, treated between January 2008 and December 2015, were retrospectively identified and included in this analysis. The study population was selected from the hospital database without prior sample size calculation, representing all eligible patients during the study period. The study included patients presenting with ischemic stroke or TIA diagnoses, with large artery atherosclerosis detected in comprehensive etiological investigations, who underwent endovascular treatment for carotid artery stenosis.

Inclusion and exclusion criteria

Patients with symptomatic or asymptomatic carotid artery stenosis (≥50%, according to the North American Symptomatic Carotid Endarterectomy Trial (NASCET) criteria [5]) were included in the study, provided they had suitable anatomy for CAS as determined by preprocedural imaging, and had undergone both pre- and post-procedural DWI (unless contraindicated). Clinical follow-up was planned according to protocol; actual follow-up and attrition are reported in the Results section.

Exclusion criteria comprised patients undergoing stenting for posterior circulation artery stenosis; patients who did not undergo post-procedural DWI due to medical instability or contraindications; patients whose follow-up data were inaccessible despite multiple contact attempts; patients with a life expectancy of less than one year due to serious comorbidity, as determined by the treating physician; and patients with absolute contraindications to MRI, including implanted devices or severe claustrophobia.

Screening and exclusions

During the study period, 46 consecutive patients were screened, and no additional cases were excluded at screening based on the pre-specified criteria; thus, the final enrolled cohort comprised 46 patients. Subsequent attrition is detailed in the Results section (8/46 (17.4%) lost to follow-up; 38 analyzed - 16 DWI+ (DWI-positive), and 22 DWI- (DWI-negative)).

CAS protocol

All procedures were performed by an experienced neurointerventional team. Clopidogrel (75 mg/day) was administered starting four to seven days prior to the procedure, and all patients were maintained on dual antiplatelet therapy with acetylsalicylic acid (ASA, 100 mg/day) and clopidogrel (75 mg/day) for at least three months post-procedure. No routine platelet function testing (e.g., ASA or P2Y12 reactivity assays) was performed during the study period; adherence was assessed by medication reconciliation. Procedures were performed under local anesthesia, with unilateral femoral artery access.

The standard protocol included vascular access through placement of a 6-8 French introducer into the common femoral artery, guide catheter placement using the exchange method over a guidewire to the external carotid artery, and use of a distal filter embolic protection device (Angioguard or Spider) in all cases. Predilatation balloon angioplasty was performed at the operator’s discretion for tight or calcified lesions to facilitate stent passage. After stent deployment with Precise or Protégé systems, post-dilatation was performed only when angiography demonstrated residual stenosis or stent under-expansion to optimize luminal gain; otherwise, it was omitted. Balloon-guide catheters with flow arrest or flow reversal were not used during the study period.

MRI protocol and image analysis

All patients underwent DWI within one to seven days before the procedure and within 24 hours after the procedure on a 1.5-T scanner (Philips Ingenia; Philips Healthcare, Best, The Netherlands). Acquisition parameters were TR/TE = 3000-4000/80-100 ms, slice thickness = 5 mm, matrix = 128 × 128, field of view = 230 × 230 mm, and b = 0 and 1000 s/mm².

All DWI studies were retrospectively reviewed side-by-side (pre- and post-procedural) by a board-certified neuroradiologist blinded to clinical outcomes.

Operational Definition and Adjudication

A new ischemic lesion required hyperintensity on b = 1000 DWI with corresponding apparent diffusion coefficient (ADC) hypointensity relative to normal-appearing white matter and absence on the baseline DWI. Foci judged to represent T2-shine-through (no ADC reduction), motion/ghosting, susceptibility or Gibbs-ringing artifacts, or noise were excluded. Morphology (punctate cortical/subcortical vs. watershed/territorial) and distribution (ipsilateral or contralateral to the treated carotid; multifocal if ≥2 discrete foci in separate territories) were recorded. The maximum axial diameter of lesions was measured for description; no formal minimum size threshold was imposed, and ambiguously small foci without unequivocal ADC reduction were not counted.

Follow-up protocol and endpoints

Patient follow-up data were retrospectively collected from medical records and included visits at 1, 3, 6, and 12 months after the procedure, then annually thereafter. Follow-up visits consisted of standardized clinical assessment, including detailed neurological examination, review of symptoms, medication compliance assessment, and neuroimaging (CT or MRI) when clinically indicated based on new symptoms or clinical deterioration.

The primary endpoint was defined as stroke or TIA occurring in any vascular territory during follow-up from the time of post-procedural DWI. Stroke was defined according to World Health Organization criteria as rapidly developing clinical signs of focal or global disturbance of cerebral function lasting more than 24 hours, or leading to death, with no apparent cause other than vascular origin. TIA was defined as a transient episode of neurological dysfunction caused by focal brain, spinal cord, or retinal ischemia without acute infarction.

Secondary endpoints included myocardial infarction, defined according to universal definition criteria; cardiovascular death from any vascular cause; major bleeding, according to International Society on Thrombosis and Haemostasis criteria; and procedural complications, including access site complications, contrast-induced nephropathy, and hemodynamic instability requiring intervention.

Statistical analysis

Statistical analyses were performed using IBM SPSS Statistics for Windows, Version 22 (Released 2013; IBM Corp., Armonk, NY, USA). Data distribution normality was evaluated using the Kolmogorov-Smirnov test with Lilliefors correction. Descriptive statistics were presented as mean ± standard deviation (SD) for continuous variables with normal distribution, median with minimum-maximum range for variables without normal distribution, and number with percentage for categorical variables.

Comparison tests included the Mann-Whitney U test for continuous variables between groups; the Chi-square test or Fisher's exact test for categorical variables, as appropriate based on expected cell frequencies; and the Kaplan-Meier method with log-rank test for survival analysis and time-to-event outcomes. A two-tailed p-value <0.05 was considered statistically significant for all analyses. All statistical tests were performed with appropriate assumptions verified prior to analysis.

## Results

Patient characteristics and demographics

A total of 46 patients were included in the final analysis after applying inclusion and exclusion criteria. The baseline demographic and clinical characteristics are summarized in Table 1. The mean patient age was 64.3 ± 7.9 years, with a predominance of male patients (35, 76.1%). The mean follow-up duration was 36.5 months, with a range of 12-60 months, providing substantial long-term outcome data.

**Table 1:** Patient Demographic Characteristics and Risk Factors Values are presented as mean ± standard deviation, or number (percentage). Statistical comparisons were performed using the Mann-Whitney U test for continuous variables and the Chi-square test for categorical variables. A p-value < 0.05 was considered statistically significant.

Characteristic	All Patients (n = 46)
Age (years), mean ± SD	64.3 ± 7.9
Male gender, n (%)	35 (76.1)
Hypertension, n (%)	35 (76.1)
Diabetes mellitus, n (%)	22 (47.8)
Hyperlipidemia, n (%)	26 (56.5)
Cardiac disease, n (%)	18 (39.1)
Cerebrovascular disease history, n (%)	17 (37.0)
Smoking, n (%)	25 (54.3)
Alcohol use, n (%)	4 (8.7)

The most prevalent cardiovascular risk factors included hypertension in 35 patients (76.1%), hyperlipidemia in 26 patients (56.5%), and diabetes mellitus in 22 patients (47.8%). A history of cardiac disease was present in 18 patients (39.1%), while previous cerebrovascular disease was documented in 17 patients (37.0%). Current smoking was reported by 25 patients (54.3%), and alcohol use was reported by four patients (8.7%).

Procedural results and DWI lesion analysis

The CAS procedure was technically successful in all 46 patients (100%), with no major complications during the immediate procedural period. Stent deployment was achieved in all cases with satisfactory angiographic results. Stents were applied to the symptomatic side in 34 patients (73.9%) and to the asymptomatic side in 12 patients (26.1%), reflecting the clinical presentation and degree of stenosis on each side.

Post-procedural DWI analysis revealed new ischemic lesions in 20 of 46 patients (43.5%) on imaging performed within 24 hours of the procedure. The majority of these lesions, specifically 19 of 20 cases (95%), remained clinically asymptomatic, with only one patient (5%) developing transient neurological symptoms corresponding to the DWI lesion location. Multiple lesions were observed in 15 of the 20 DWI-positive patients (75%), indicating that, when embolic events occur, they frequently involve multiple cerebral territories.

Post-procedural hypotension, defined as systolic blood pressure less than 90 mmHg lasting more than 30 minutes despite adequate fluid resuscitation and vasopressor support, occurred in 15 patients (32.6%). This complication was managed successfully in all cases with appropriate medical intervention, and no patient experienced persistent hemodynamic compromise.

Group comparison according to DWI lesion status

Patients were stratified into two groups based on the presence of new DWI lesions: DWI+ (n = 20) and DWI- (n = 26) groups. The detailed comparison of baseline characteristics between these groups is presented in Table 2. Statistical analysis revealed no significant differences between the groups in terms of demographic characteristics, vascular risk factors, incidence of post-procedural hypotension, or laterality of stenting (all p-values > 0.05).

**Table 2:** Group Comparison According to DWI Lesion Presence Values are presented as mean ± standard deviation, or number (percentage). Statistical comparisons were performed using the Mann-Whitney U test for continuous variables, and the Chi-square test or Fisher's exact test for categorical variables. A p-value < 0.05 was considered statistically significant. DWI: diffusion-weighted imaging; CVD: cerebrovascular disease

Characteristic	DWI- (n = 22)	DWI+ (n = 16)	p-value
Age (years), mean ± SD	64.1 ± 8.2	64.6 ± 7.5	0.842
Male gender, n (%)	18 (81.8)	11 (68.8)	0.396
Hypertension, n (%)	17 (77.3)	12 (75.0)	0.880
Diabetes mellitus, n (%)	10 (45.5)	8 (50.0)	0.796
Hyperlipidemia, n (%)	13 (59.1)	9 (56.2)	0.855
Cardiac disease, n (%)	10 (45.5)	5 (31.2)	0.266
CVD history, n (%)	8 (36.4)	6 (37.5)	0.809
Smoking, n (%)	12 (54.5)	9 (56.2)	0.938
Post-procedural hypotension, n (%)	8 (36.4)	5 (31.2)	0.741
Symptomatic side stenting, n (%)	16 (72.7)	12 (75.0)	0.883

The mean age was similar between groups (DWI-: 64.1 ± 8.2 years vs. DWI+: 64.6 ± 7.5 years, p = 0.842). Male gender distribution was comparable (DWI-: 18 (81.8%) vs. DWI+: 11 (68.8%), p = 0.396). Cardiovascular risk factors showed no significant differences, including hypertension (DWI-: 17 (77.3%) vs. DWI+: 12 (75.0%), p = 0.880), diabetes mellitus (DWI-: 10 (45.5%) vs. DWI+: 8 (50.0%), p = 0.796), and hyperlipidemia (DWI-: 13 (59.1%) vs. DWI+: 9 (56.2%), p = 0.855).

Long-term follow-up results

**Figure 1:**
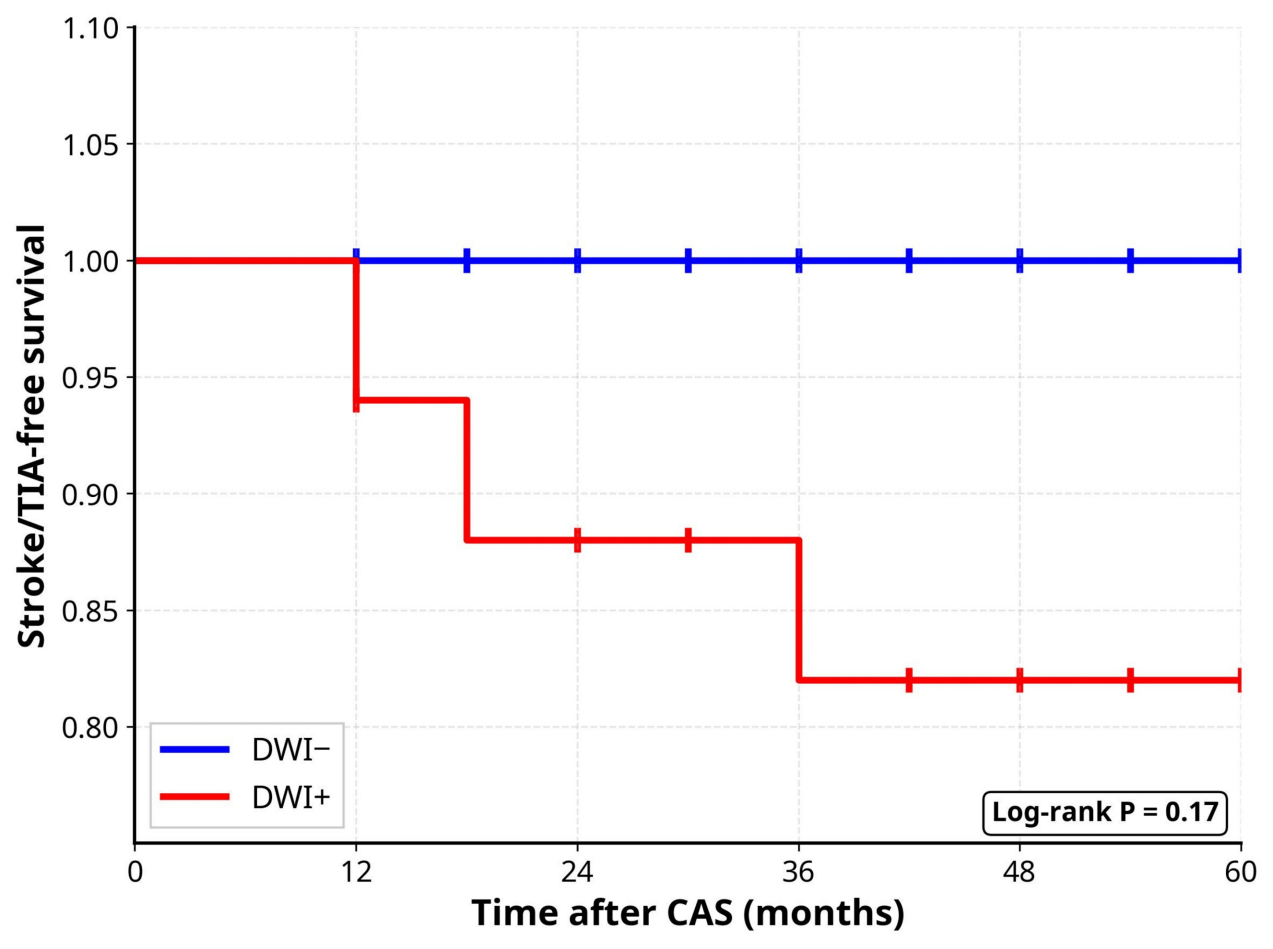
Kaplan-Meier Curves for Stroke/TIA-Free Survival After CAS Stratified by Post-procedural DWI Status DWI+ indicates the presence of new ischemic lesions on post-procedural diffusion-weighted imaging (n = 16; events = 2); DWI-, the absence of new lesions (n = 22; events = 0). Log-rank p = 0.17. Censoring tick marks indicate the last follow-up. Axes: x = months after CAS; y = stroke/TIA-free survival. DWI: diffusion-weighted imaging; CAS: carotid artery stenting; TIA: transient ischemic attack

Eight patients (17.4%) were lost to follow-up. Data from 38 patients were analyzed (16 DWI+ and 22 DWI-). Regarding the primary endpoint of stroke/TIA recurrence, two patients (12.5%) in the DWI+ group experienced stroke recurrence, while no recurrences were observed in the DWI- group. The difference was not statistically significant (p = 0.170). Kaplan-Meier survival analysis demonstrated no significant difference in stroke-free survival between groups (log-rank p = 0.17) (Figure 1).

Secondary endpoints revealed myocardial infarction in two patients (12.5%) in the DWI+ group versus three patients (13.6%) in the DWI- group (p = 1.000). Total vascular events (stroke + myocardial infarction) occurred in four patients (25.0%) in the DWI+ group versus three patients (13.6%) in the DWI- group (p = 0.639). No cases of major bleeding or cardiovascular death were observed in either group during follow-up.

Procedural safety

No procedure-related death or major stroke occurred during the immediate procedural period. No patient experienced stroke, myocardial infarction, or death during the first 30 days following the procedure, meeting established safety benchmarks for carotid intervention. These findings indicate that CAS can be safely performed in experienced centers with appropriate patient selection and procedural expertise.

Minor complications included transient access site hematoma in two patients (4.3%) and contrast-induced nephropathy in one patient (2.2%), all of which resolved without long-term sequelae. The low complication rate reflects the experience of the intervention team and adherence to established procedural protocols.

## Discussion

Main findings

This retrospective cohort study investigated the effect of new ischemic lesions detected by DWI following CAS on long-term stroke recurrence in a Turkish patient population. The principal findings demonstrate that new DWI lesions developed in 20 of 46 patients (43.5%) following CAS, which is consistent with previously reported rates in the international literature. During a mean follow-up period of 36.5 months, stroke recurrence occurred in 2 of 16 patients (12.5%) in the DWI+ group, while no recurrence was observed in the 22 patients of the DWI- group. Although this difference did not achieve statistical significance (p = 0.170), the findings suggest that DWI lesions may serve as potential risk markers for long-term cerebrovascular events and warrant further investigation in larger cohorts.

The clinical significance of these findings may extend beyond the immediate procedural period and may inform future approaches to long-term patient management and risk stratification; however, these observations are hypothesis-generating. The absence of statistical significance may be attributed to the relatively small sample size and the low overall event rate, which is consistent with the generally favorable long-term prognosis following successful carotid revascularization in contemporary practice.

Comparison with previous studies

Our findings are partially consistent with previous reports. The incidence of new DWI lesions (43.5%) falls within the range reported in previous investigations (30%-70%) [14,15]. In the ICSS-MRI substudy, Gensicke et al. reported a five-year recurrent stroke/TIA risk of 22.8% in DWI+ patients and 8.8% in DWI- patients [16]. In our study, recurrence rates were lower (DWI+: 12.5% and DWI-: 0%). This discrepancy may be explained by the smaller sample size, differences in follow-up duration, and patient demographics.

In contrast, the long-term follow-up study published by Donners et al. in 2023 aligns more closely with our findings [17]. That study demonstrated that DWI lesions were not associated with long-term stroke risk after 12.5 years of follow-up.

Clinical implications

Our results are hypothesis-generating rather than practice-changing. A numerically higher recurrence rate was observed in DWI+ patients, who might merit closer clinical vigilance. Guideline-concordant secondary prevention should be prioritized - e.g., statin intensification and aggressive control of modifiable vascular risk factors. Antiplatelet regimen review and adherence assessment may be considered on an individual basis; however, because routine platelet function testing was not performed in this cohort, any inference about antiplatelet adjustment remains exploratory. Post-procedural DWI may provide prognostic information; however, its routine use and impact on management require validation in larger, adequately powered cohorts.

Strengths and limitations

Strengths of this study include its retrospective inclusion of consecutive patients, use of standardized protocols, long-term follow-up (mean 36.5 months), and performance in an experienced center. Limitations include the small sample size (46 patients), single-center design, loss to follow-up (17.4%), and limited DWI lesion characterization (e.g., no lesion volumetry; no lesion count or distribution reporting - including hemispheric laterality and vascular territory; and no perfusion or quantitative ADC correlation). No a priori sample size calculation was performed; the modest sample and low event rate limit statistical power and raise the possibility of a type II error. With only two primary events, multivariable adjustment was not feasible; therefore, residual confounding by age and comorbidities cannot be excluded, and age-related/new small-vessel DWI lesions unrelated to carotid stenosis remain a possibility despite our requirement that lesions be new versus baseline and detected within 24 hours after CAS. The 17.4% loss to follow-up may bias time-to-event estimates if censoring is informative; Kaplan-Meier analyses assume non-informative censoring, which cannot be guaranteed in this retrospective cohort. Accordingly, effect estimates should be interpreted with caution. Additionally, routine platelet function testing was not performed, precluding assessment of high on-treatment platelet reactivity as a contributor to new DWI lesions. Criteria for post-dilatation were operator-dependent, and balloon-guide catheters with flow arrest/flow reversal were not used; this may influence embolic risk and limit generalizability to contemporary practice. Furthermore, while our study focused on recurrent clinical stroke/TIA, we did not evaluate longer-term outcomes such as chronic ischemic changes on follow-up imaging or potential contributions to vascular cognitive impairment. These aspects represent important avenues for future research.

Future directions

Future research should validate these findings through multicenter studies with larger cohorts. Randomized controlled trials, comparing different antiplatelet regimens in DWI+ patients, are warranted. Investigation of advanced imaging modalities, and biomarker-based risk stratification, should be explored.

## Conclusions

This retrospective cohort study demonstrated that new DWI lesions developing after CAS may serve as potential risk markers for long-term stroke recurrence, although statistical significance was not achieved in this relatively small cohort. The findings suggest a trend toward increased cerebrovascular risk in patients with post-procedural DWI lesions, with stroke recurrence occurring in 12.5% of DWI+ patients, compared to 0% in DWI- patients during a mean follow-up of 36.5 months.

To the best of our knowledge, our study provides the first comprehensive long-term data on this subject in the Turkish population, contributing to the growing body of international literature on this important topic. However, the relatively small sample size and single-center design should be considered when interpreting these results. Validation through larger, multicenter studies with extended follow-up periods is necessary to confirm these preliminary findings and establish definitive clinical guidelines for the management of patients with post-procedural DWI lesions.
